# Circulating Galectin-3 and Aldosterone for Predicting Atrial Fibrillation Recurrence after Radiofrequency Catheter Ablation

**DOI:** 10.1155/2022/6993904

**Published:** 2022-05-23

**Authors:** Zhong-bao Ruan, Run-feng Gao, Fei Wang, Ge-cai Chen, Jun-guo Zhu, Yin Ren, Li Zhu

**Affiliations:** ^1^Department of Cardiology, Jiangsu Taizhou People's Hospital, Taizhou 225300, China; ^2^Dalian Medical University, Dalian 116044, China

## Abstract

**Background:**

Circulating galectin-3 (Gal-3) and aldosterone (ALD) are involved in fibrosis and inflammation. However, their potential value as predictors of atrial fibrillation (AF) recurrence after radiofrequency catheter ablation (RFCA) is unknown or controversial. Therefore, the aim of this study was to assess the relationship between baseline Gal-3, ALD levels, and AF recurrence in patients performing RFCA.

**Methods:**

153 consecutive patients undergoing RFCA were included. Gal-3 and ALD were measured at baseline. Univariate and multivariate Cox regressions were performed to determine the predictors of AF recurrence. Receiver operating characteristic (ROC) curve and Kaplan-Meier (K-M) curve were used to assess the value of predictors.

**Results:**

There were 35 (22.88%) cases of AF recurrence after RFCA. The recurrence group had significantly higher preoperative serum levels of Gal-3 and ALD than the nonrecurrence group. Univariate and multivariate analysis showed that Gal-3 (HR = 1.28, 95% CI: 1.04-1.56, *p* = 0.02) and ALD (OR = 1.02, 95% CI: 1.00-1.03, *p* < 0.03) were significantly associated with AF recurrence after RFCA. The area under the curve (AUC) of preoperative serum Gal-3, ALD, and 2 combined to predict the recurrence of AF patients after RFCA was 0.636, 0.798, and 0.893, respectively, while sensitivity was 65.32%, 71.69%, and 88.61%, respectively and specificity was 77.46%, 78.53%, and 86.0%, respectively. Patients with Gal-3 above the cutoff value of 14.57 pg/ml had higher frequent AF recurrence than the patients with Gal − 3 ≤ 14.57 pg/ml (35% vs. 12%, *p* < 0.001) during a follow-up. Meanwhile, patients with ALD above the cutoff value of 243.61 pg/ml also had a higher AF recurrence rate than those with ALD ≤ 243.61 pg/ml (37% vs. 11%, *p* < 0.001) during a follow-up. The recurrence rate in patients with Gal − 3 > 14.57 pg/ml + ALD > 243.61 pg/ml was higher than that in patients with baseline Gal − 3 > 14.57 pg/ml or ALD > 243.61 pg/ml and patients with Gal − 3 ≤ 14.57 pg/ml + ALD ≤ 243.61 pg/ml (57% vs. 14% vs. 9%, *p* < 0.01, respectively).

**Conclusion:**

AF recurrence after RFCA had higher baseline Gal-3 and ALD levels, and higher preoperative circulating Gal-3 and ALD levels were independent predictors of AF recurrence for patients undergoing RFCA, while combination of preoperative Gal-3 and ALD levels has higher prediction accuracy.

## 1. Background

Atrial fibrillation (AF) is the most common abnormal cardiac rhythm disease occurring in nearly 3% of the adult population, and all-cause mortality is twice as high as in non-AF patients [1]. Catheter ablation has been recommended for patients with AF who are resistant or unwilling to antiarrhythmic drugs [2]. The onset and development of AF are often accompanied by atrial structural remodeling, characterized primarily by left atrial inflammation and fibrosis, which has been found to be strongly associated with AF recurrence after ablation [3, 4]. In this regard, activation of nucleotide-binding and oligomerization domain- (NOD-) like receptor (NLRP3) inflammasome is associated with linked to the pathogenesis of cardiovascular disease (CVD) such as AF [5, 6]. Polymorphisms of the 5-10-methylenetetrahydrofolate reductase (MTHFR) gene, which plays key roles in cellular homeostasis, are also associated with various diseases including AF [7, 8].

Galectin-3 (Gal-3), a *β*-galactoside-binding lectin secreted primarily by activated macrophages, plays a variety of functions in cellular processes such as inflammation, apoptosis, angiogenesis, adhesion, and migration [9]. Meanwhile, Gal-3 associates with a variety of fibrosis-related diseases, such as liver cirrhosis, lung fibrosis, and cardiac fibrosis during heart failure [10, 11]. It has been shown that increased Gal-3 levels correlate with atrial fibrosis [12] and are related to AF recurrence after ablation [13–15]. However, another studies evaluating the association between preprocedural circulating Gal-3 levels and the recurrence of AF after catheter ablation showed inconsistent results [16, 17]. Aldosterone (ALD), reported to induce fibrotic changes in the myocardium, is associated with systemic inflammation and fibrosis in patients with AF. Antifibrotic medication may reduce the fibrosis in the myocardium and prevent the occurrence of AF [18, 19].

Previous studies have also demonstrated that ALD-activated macrophages play a major role in cardiac myofibrosis by promoting the secretion of Gal-3 and Gal-3 was required for the inflammatory and fibrotic reactions induced by ALD, which suggest that there is an interaction between Gal-3 and aldosterone in myocardial fibrosis [20, 21]. However, there are few studies to discuss the preoperative plasma ALD as a predictive biomarker of AF recurrence after catheter ablation. At the same time, there are few relevant studies focusing on the combined predictive value of preoperative Gal-3 and ALD levels for AF recurrence after radiofrequency catheter ablation (RFCA).

For the above reasons, there is a growing interest for a further analysis of Gal-3 and ALD as predictors of AF recurrence after RFCA. Therefore, the aim of this study was to assess the relationship between Gal-3, ALD levels, and recurrence after catheter ablation in patients with nonvalvular AF and evaluate the predictive value of preoperative plasma Gal-3 combined with ALD.

## 2. Materials and Methods

### 2.1. Study Population

From July 2020 to July 2021, a total of 153 AF patients who met the criteria of 2020 ESC diagnostic and management guidelines [22] and underwent RFCA at Jiangsu Taizhou People's Hospital were selected. Those with age less than 18 years, valvular AF, severe heart failure (NYHA III or IV), left atrium or left atrial appendage thrombosis, and long-term administration of anti-inflammatory drugs, ACEI/ARB, spironolactone, and other antagonistic aldosterone drugs were excluded. All patients underwent routine preoperative transesophageal echocardiography (TEE) or dual-source coronary CT angiography (CTA) to exclude intra-atrial thrombus. Left atrial diameter (LAD), end-diastolic left ventricular diameter (LVDD), end-systolic left ventricular diameter (LVDS), and left ventricular ejection fraction (LVEF) were measured by echocardiography. The study was approved by the Ethics Committee of Jiangsu Taizhou People's Hospital. Written informed consent was obtained from all patients before the procedure.

### 2.2. RFCA Procedure

Noninvasive arterial blood pressure, electrocardiogram, and oxygen saturation were monitored during the whole procedure. Right femoral vein and left femoral vein were entered using Seldinger technique. The left atrium and pulmonary vein models were constructed with CARTO three-dimensional mapping system (CARTO R3; Biosense Webster, Irvine, CA, USA). Pulmonary vein isolation (PVI) was performed in all patients with Cool Flex catheters. Endpoint of PVI was defined as the block of exit and entrance. For persistent atrial fibrillation, left atrial substrate mapping and homogenous ablation of low-voltage areas were performed. Potentials with amplitudes over 0.5 mV were defined as normal and potentials under 0.2 mV as low voltage. After ablation, antiarrhythmic drugs and oral anticoagulation were used for 3 months. Proton pump inhibitors were added for 4 weeks.

### 2.3. Gal-3 and ALD Measurements

Blood samples obtained before catheter ablation from peripheral vein were centrifuged (3000 revolutions, 10°C, 5 min) and stored at -80°C until time for analysis. The levels of serum Gal-3 and ALD were determined by enzyme-linked immunosorbent assay (ELISA) using Gal-3 reagent kit (MEIMIAN, Guangzhou Scissors Hand Technology) and ALD reagent kit (SinoBestBio, Guangzhou Scissors Hand Technology), respectively. Interassay coefficient of variation for ELISA assay was defined as less than 5%, and intra-assay variance was <5%.

### 2.4. Postoperative Follow-Up

A follow-up was performed for all patients in the outpatient clinic for 6 months after RFCA. Electrocardiography (ECG) and 24 h Holter recordings were obtained at 3 and 6 months after catheter ablation. During every follow-up period, patients were encouraged to visit a physician when symptomatic. AF recurrences were defined as any atrial tachyarrhythmia lasting >30 s documented by ECG or 24 h Holter monitoring after the third month postablation.

### 2.5. Statistical Analysis

SPSS 26.0 statistical software was used to perform the data analysis. Continuous variables were expressed as the mean ± standard deviation (mean ± SD), and the *t*-test was used for comparison. Categorical variables were expressed as the number and percentage (*n*, %) and compared using the chi-square test or Fisher's exact test. The risk factors for AF recurrence after catheter ablation were analyzed by Cox regression model. The ROC curve was used to evaluate the predictive value of Gal-3 and ALD for AF recurrence after catheter ablation. Kaplan-Meier survival curves were plotted, and the outcomes were compared between groups using the log-rank test.

## 3. Results

### 3.1. Comparison of Clinical Data between the Recurrence Group and the Nonrecurrence Group

A total of 153 AF patients were enrolled, and the baseline characteristics are detailed in Table 1. Mean age of the patients was 60.13 ± 5.142 years, and 83 (54.25%) were males. 78 (50.98%) patients had paroxysmal AF (Px-AF) and 75 (49.02%) had persistent AF (Ps-AF). As shown in Table 1, there were 35 (22.88%) cases of AF recurrence after RFCA (recurrence group), in which 12 cases with Px-AF and 23 cases with Ps-AF, while 118 (77.12%) without AF recurrence (the nonrecurrence group). The comparison between the recurrence group and the nonrecurrence group showed a statistically significant difference in age, type of AF, and LAD. The recurrence group had significantly higher serum levels of Gal-3 and ALD than the nonrecurrence group.

### 3.2. Correlation between Gal-3, ALD, and AF Recurrence

On univariate Cox analysis, higher of age, Ps-AF, LAD, Gal-3, and ALD were associated with AF recurrence, whereas in multivariate analysis, only Gal-3 (HR = 1.28, 95% CI: 1.04-1.56, *p* = 0.02) and ALD (OR = 1.02, 95% CI: 1.00-1.03, *p* < 0.03) remained significantly associated with AF recurrence after RFCA (Table 2).

### 3.3. The Predictive Value of Preoperative Gal-3 and ALD Levels for AF Recurrence after RFCA Analyzed by ROC

**Figure 1:**
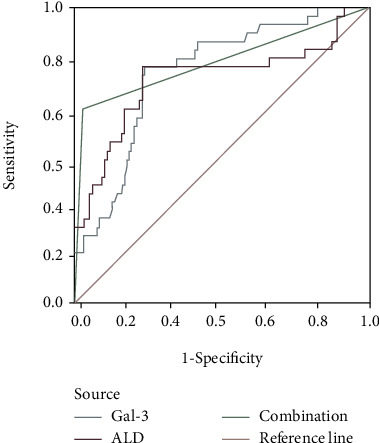
Analysis of preoperative Gal-3, ALD, and 2 combined for predicting AF recurrence after RFCA with ROC curve.

As shown in Table 3 and Figure 1, ROC analysis results showed that the cutoff values of Gal-3 and ALD levels were 14.57 pg/ml and 243.61 pg/ml, respectively. The area under the curve (AUC) of preoperative serum Gal-3, ALD, and 2 combined to predict the recurrence of AF patients after RFCA was 0.636, 0.798, and 0.893, respectively. The sensitivity was 65.32%, 71.69%, and 88.61%, respectively. The specificity was 77.46%, 78.53%, and 86.0%, respectively. The Youden indexes were 0.44, 0.52, and 0.86, respectively.

### 3.4. Freedom of AF Recurrence after RFCA Stratified for Preoperative Gal-3, ALD Levels, and 2 Combined

**Figure 2:**
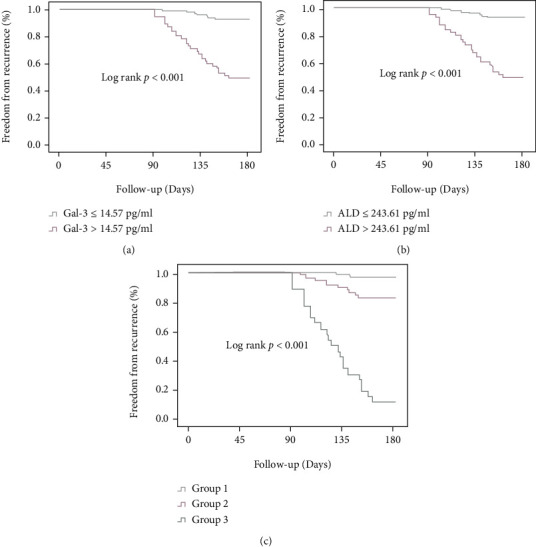
Freedom of AF recurrence after RFCA stratified for preoperative Gal-3, ALD, and 2 combined. (a) Recurrence of AF by preoperative Gal-3 levels. (b) Recurrence of AF by preoperative ALD levels. (c) Recurrence of AF by preoperative Gal-3 combined with ALD. Group 1 for patients with Gal − 3 ≤ 14.57 pg/ml + ALD ≤ 243.61 pg/ml, group 2 for patients with baseline Gal − 3 > 14.57 pg/ml or ALD > 243.61 pg/ml, and group 3 for patients with Gal − 3 > 14.57 pg/ml + ALD > 243.61 pg/ml.

Patients with Gal-3 above the cutoff value of 14.57 pg/ml had higher frequent AF recurrence than patients with Gal − 3 ≤ 14.57 pg/ml (35% vs. 12%, *p* < 0.001) during a follow-up (Figure 2(a)). Patients with ALD above the cutoff value of 243.61 pg/ml also had a higher AF recurrence rate than those with ALD ≤ 243.61 pg/ml (37% vs. 11%, *p* < 0.001) during a follow-up (Figure 2(b)). When the combined Gal-3 and ALD assay was performed, the patients were divided into three groups according to the cutoff value of preoperative Gal-3 and ALD levels: group 1 for patients with Gal − 3 ≤ 14.57 pg/ml + ALD ≤ 243.61 pg/ml (*n* = 37, 24%), group 2 for patients with baseline Gal − 3 > 14.57 pg/ml or ALD > 243.61 pg/ml (*n* = 72, 47%), and group 3 for patients with Gal − 3 > 14.57 pg/ml + ALD > 243.61 pg/ml (*n* = 37, 24%). The K-M survival analysis of the three groups is shown in Figure 2(c). The recurrence rate of group 3 was higher than that of group 2 and group 1 (57% vs. 14% vs. 9%, *p* < 0.01, respectively).

## 4. Discussion

In recent years, studies have demonstrated that the Gal-3 and RAAS systems are strongly associated with AF [12–15]. Another studies suggested that serum Gal-3 was not associated with sinus rhythm maintenance and AF recurrence after RFCA [16, 17]. The results of this study confirmed that (1) the levels of Gal-3 and ALD were significantly higher in patients with AF than those without AF; (2) preoperative high Gal-3 and ALD levels were independent predictors of short-term recurrence in AF patients after RFCA; (3) the combined predictive value of Gal-3 and ALD was higher than the single. To the best of our knowledge, the present study is the first to investigate the combined predictive value of Gal-3 and ALD in AF recurrence after RFCA.

Atrial remodeling of AF, which includes structural remodeling and electrical remodeling, is characterized by myocardial cell hypertrophy, myocardial fibrosis, and abnormal expression of cell-to-cell or cell-to-matrix, in which myocardial fibrosis is a hallmark and plays an important role in AF pathogenesis [23]. The inflammatory response is an important signaling pathway for myocardial fibrosis.

As an inflammatory marker, the MTHFR polymorphisms are reported to be coupled with aberrant DNA methylation and various inflammatory diseases [7, 8]. As one of the most important inflammasomes and one of the early biomarkers of periodontitis, the activation of NLRP3 inflammasome is associated with the facilitated cardiac fibroblasts and the AF development [5, 6]. As a member of the galectins family, Gal-3 is an inflammatory factor secreted by activated macrophages, mast cells, eosinophils, and neutrophils [24]. Gal-3 mediates electrical and structural remodeling during AF progression by promoting fibroblast activation and differentiation, followed by myocardial fibrosis, remodeling, cell dysfunction, and ultimately AF [25]. Reports showed that the Gal-3 inhibitor GMCT reduced atrial fibroblast proliferation and mitigated both electrical and structural remodeling during AF progression [26]. Increased Gal-3 levels are also found to be related with AF recurrence after ablation [13–15], but there is a controversy [16, 17]. In this study, baseline Gal-3 levels in the recurrence group were higher than those in the nonrecurrence group. Univariate Cox analysis showed that higher levels of Gal-3 were associated with AF recurrence after RFCA. Patients with Gal-3 above the cutoff value of 14.57 pg/ml had higher frequent AF recurrence than patients with Gal − 3 ≤ 14.57 pg/ml during a follow-up. Our results demonstrate that preoperative Gal-3 levels may predict the AF recurrence after RFCA, which is consistent with the results of previous studies [13–15].

Similarly, the RAAS is another important signaling pathway for myocardial fibrosis and participates in atrial electrophysiological and structural remodeling during AF development [27]. ALD can cause atrial arrhythmias characterized by atrial fibrosis, cardiomyocyte hypertrophy, and conduction disorders. ALD receptor antagonists can be used to reduce the level of ALD in patients with AF and inhibit atrial fibrosis, thus reduce the occurrence and persistence of AF [28]. In the present study, baseline ALD levels in the recurrence group were also higher than those in the nonrecurrence group. Univariate Cox analysis showed that higher levels of ALD were associated with AF recurrence after RFCA. Patients with ALD above the cutoff value of 243.61 pg/ml also had a higher AF recurrence rate than those with ALD ≤ 243.61 pg/ml during a follow-up. Our results suggest that preoperative ALD levels may predict the AF recurrence after RFC.

At the same time, there are few relevant studies focusing on the combined predictive value of preoperative Gal-3 and ALD levels for AF recurrence after RFCA. In this study, ROC analysis results showed that the AUC, sensitivity, specificity, and Youden index of combination with Gal-3 and ALD to predict the recurrence of AF patients after RFCA were 0.893, 88.61%, 86.0%, and 0.86, respectively. The K-M survival analysis according to the cutoff value of preoperative Gal-3 and ALD levels shows that patients with Gal − 3 > 13.60 pg/ml and ALD > 198.43 pg/ml have relatively higher predictive value for AF recurrence after RFCA. The results suggest that combination of preoperative Gal-3 and ALD levels has high prediction accuracy and can be greatly used to predict the recurrence risk of AF in patients after RFCA.

There are some limitations in the present study. Firstly, some AF patients have the comorbidities that may affect myocardial fibrosis and remodeling, such as hypertension, diabetes, and coronary heart disease, which may lead to inaccurate measurements of serum levels of Gal-3 and ALD. Secondly, statins, used in some patients in this study, have been reported to alter Gal-3 concentrations in humans [29], which may have an impact on measures. Thirdly, Gal-3 and ALD were measured at baseline, while the levels of Gal-3 and ALD during a 6-month follow-up were not detected, so the changes of Gal-3 and ALD between preoperative and postoperative on AF recurrence were not explored.

## 5. Conclusions

The present study indicated that patients with AF recurrence after RFCA had higher baseline Gal-3 and ALD levels. At the same time, higher preoperative circulating Gal-3 and ALD levels were independent predictors of AF recurrence for patients undergoing RFCA, while combination of preoperative Gal-3 and ALD levels has higher prediction accuracy.

## Figures and Tables

**Table 1:** Baseline characteristics of the study population.

Clinical parameters	All (*n* = 153)	Recurrence (*n* = 35)	Nonrecurrence (*n* = 118)	*p* value
Sex (*n*, %)				
Male	83 (54.25)	17 (48.57)	66 (55.93)	
Female	70 (45.75)	18 (51.43)	52 (44.07)	0.28
Age (years)	60.13 ± 5.142	61.80 ± 4.30	59.63 ± 5.55	0.04
BMI (kg/m^2^)	22.54 ± 1.96	22.14 ± 1.67	22.48 ± 2.49	0.45
GFR (ml/min/1.73 m^2^)	89.42 ± 8.02	89.14 ± 8.48	89.58 ± 7.87	0.77
Type of AF, *n* (%)				
Px-AF	78 (50.98)	12 (34.29)	66 (55.93)	0.02
Ps-AF	75 (49.02)	23 (65.71)	52 (44.07)	
CHA2DS2-VASc score	2.63 ± 0.84	2.66 ± 0.87	2.58 ± 0.83	0.62
As-BLED score	1.84 ± 0.73	1.80 ± 0.76	1.89 ± 0.66	0.50
Smoking history, *n* (%)	30 (19.61)	7 (20.00)	23 (19.49)	0.56
Hypertension, *n* (%)	47 (30.72)	11 (31.43)	36 (30.51)	0.54
Diabetes, *n* (%)	25 (16.34)	6 (17.14)	19 (16.10)	0.53
Coronary heart disease, *n* (%)	38 (24.84)	9 (25.71)	29 (24.58)	0.53
LVEF (%)	63.93 ± 5.53	64.26 ± 5.15	63.49 ± 5.86	0.49
LAD (mm)	40.75 ± 4.06	41.94 ± 3.95	40.14 ± 4.03	0.02
LVDD (mm)	49.04 ± 3.52	49.03 ± 3.73	49.04 ± 3.45	0.77
LVDS (mm)	30.92 ± 3.45	30.74 ± 3.66	31.02 ± 3.38	0.65
Use of medications				
Statins	42 (27.45)	10 (28.57)	32 (27.12)	0.51
Beta blockers	45 (29.41)	11 (31.43)	34 (28.81)	0.46
Gal-3 (pg/ml)	14.52 ± 1.91	14.97 ± 1.86	13.91 ± 1.88	0.004
ALD (pg/ml)	230.65 ± 26.61	241.38 ± 30.30	227.50 ± 24.91	0.007

**Table 2:** Clinical parameters associated with AF recurrence by univariate and multivariate Cox analysis.

Clinical parameters	Univariate analysis			Multivariate analysis		
	HR	95% CI	*p* value	HR	95% CI	*p* value
Age	1.07	1.01-1.05	0.03	1.06	0.99-1.13	0.09
Ps-AF	2.23	1.11-4.49	0.02	1.82	0.87-3.80	0.11
LAD (mm)	1.10	1.01-1.19	0.02	1.05	0.98-1.14	0.19
Gal-3 (pg/ml)	1.35	1.10-1.65	0.003	1.28	1.04-1.56	0.02
ALD (pg/ml)	1.02	1.01-1.04	0.006	1.02	1.00-1.03	0.03

**Table 3:** ROC analysis of preoperative Gal-3 and ALD levels for AF recurrence after RFCA.

	Cutoff value	AUC	Sensitivity (%)	Specificity (%)	Youden index	95% CI	*p* value
Gal-3	14.57 pg/ml	0.636	65.32	77.46	0.44	0.563-0.740	0.02
ALD	243.61 pg/ml	0.798	71.69	78.53	0.52	0.725-0.843	0.003
Combined	—	0.893	88.61	86.0%	0.86	0.853-0.924	0.001

## Data Availability

The data used to support the findings of this study are available from the corresponding author upon request.
